# Evaluation of the clinical performance of the HISCL‐5000 analyzer in the detection of Krebs von den Lungen‐6 antigen and its diagnostic value in interstitial lung disease

**DOI:** 10.1002/jcla.23070

**Published:** 2019-11-06

**Authors:** Jingxian Wang, Zhifeng Huang, Mingshan Xue, Huimin Huang, Xiaomao Zheng, Nanshan Zhong, Baoqing Sun

**Affiliations:** ^1^ Department of Pathophysiology Guizhou Medical University Guiyang China; ^2^ Department of Allergy and Clinical Immunology Guangzhou Institute of Respiratory health State Key Laboratory of Respiratory Disease National Clinical Research Center of Respiratory Disease First Affiliated Hospital of Guangzhou Medical University Guangzhou China; ^3^ Experiment Center of Stem Cell and Tissue Engineering Research Guizhou Medical University Guiyang China

**Keywords:** agreement, comparison, HISCL‐5000, interstitial lung disease, KL‐6, LUMIPULSE G1200

## Abstract

**Background:**

The sputum saccharide chain antigen (Krebs von den Lungen‐6 [KL‐6]) is a serum biomarker of lung injury. We aimed to evaluate the clinical performance of the automated immunoassay analyzer HISCL‐5000 in detecting KL‐6 by comparing it with LUMIPULSE G1200 and determine the diagnostic value of KL‐6 in interstitial lung disease (ILD).

**Methods:**

A total of 145 serum samples from patients were tested using the two automated immunoassay analyzers in parallel.

**Results:**

With a cutoff level of 500 U/mL, comparing the two systems, the agreement, sensitivity, specificity, and kappa value were 99.20%, 100%, 98.63%, and 0.984 (95% CI, 0.952‐1.000), respectively. Spearman's correlation and ICC showed that there was a strong correlation between serum KL‐6 levels measured by the two systems (*r*_S_ = .991 [95% CI, 0.981‐0.995], ICC = 0.984 [95% CI, 0.978‐0.989], *P* < .01). The clinical diagnosis agreement rate in both systems was >80%. The kappa value was 0.707 (95% CI, 0.582‐0.832; SYSTEM B) and 0.707 (95% CI, 0.588‐0.826; SYSTEM A). The KL‐6 level in the ILD group (1339.5, 662.5‐2363) was significantly higher than that in the non‐ILD groups (252, 158.5‐353; Mann‐Whitney *U* = 381.5, *P* < .01), and the KL‐6 level (1558, 726‐2772.5) in the ILD group detected by SYSTEM A was significantly higher than that in the lung cancer group (339, 207‐424), other respiratory disease group (249, 194‐366), and control group (198, 131.5‐297; Kruskal‐Wallis *H* = 63.19, *P* < .01).

**Conclusions:**

HISCL‐5000 showed well‐concordant results with those of HISCL‐5000 in the KL‐6 tests. In patients with ILD, KL‐6 showed a good diagnostic performance.

## 1 INTRODUCTION

Interstitial lung disease (ILD) is a group of diseases characterized by various forms of pulmonary interstitial inflammation and fibrosis, which are usually chronic, progressive, and fatal,^1^ causing death 2‐5 years after diagnosis in most patients.^2^, ^3^ These disorders primarily affect the pulmonary interstitium, alveolar cavity, and bronchioles.^4^ Presently, clinical diagnostic methods of ILD are extremely limited, including HRCT, pulmonary function test, bronchial lavage, lung biopsy, and other examinations, which require specific medical equipment and will lead to patient discomfort.^5^ Therefore, it is necessary to find safe, simple, and reproducible biological markers for the prediction and early diagnosis of ILD.^6^

Since the most important feature of ILD is repeated damage or repair of type II alveolar epithelial cells, Krebs von den Lungen‐6 (KL‐6) secreted by type II alveolar epithelial cells is highly regarded.^6^, ^7^ When epithelial cells are damaged, KL‐6 enters the circulation, promotes fibroblast proliferation and migration, inhibits apoptosis, and aggravates the development of pulmonary fibrosis. Therefore, KL‐6 is considered the most accurate biomarker in the diagnosis of ILD.^8^, ^9^ Studies have shown that, when KL‐6 has a cutoff value of 500 U/mL, it can distinguish among patients with ILD, healthy subjects, and those with other benign non‐ILDs.^10^

Enzyme‐linked immunosorbent assay using an anti‐KL‐6 monoclonal antibody has been widely used in clinical laboratories.^11^ Fully automated analyzers using various methodologies, such as chemiluminescent microparticle immunoassay (CMIA) or chemiluminescent enzyme immunoassay (CLEIA), for example, LUMIPULSE G1200 (Fujirebio Diagnostics), had been introduced and used clinically. Robust midsized fully automated chemiluminescence‐based enzyme immune‐analyzers and their analytical performances have been evaluated.^12^, ^13^ Recently, Sysmex Corporation has released a newly developed KL‐6 assay kit using the HISCL‐5000 analyzer.

This study used the HISCL‐5000 analyzer (hereinafter referred to as SYSTEM A) and LUMIPULSE G1200 analyzer (hereinafter referred to as SYSTEM B) to measure serum KL‐6 levels in patients with ILD, lung cancer, and other respiratory diseases and healthy individuals. This study aimed to investigate the diagnostic value of serum KL‐6 in ILD and evaluate the clinical performance of the HISCL‐5000 analyzer.

## 2 MATERIALS AND METHODS

### 2.1 Information of materials

This is a retrospective observational study. We collected serum samples from 145 individuals between May 2018 and October 2018 at The First Affiliated Hospital of Guangzhou Medical University. Of 145 subjects, 25 had lung cancer, 56 had ILD, 35 had other respiratory diseases, and 29 were healthy individuals (control group) who underwent regular health checkup. There were 83 (57.24%) men and 62 women with an age distribution of 56 years (46, 67; median [IQ]). The general characteristics of the cohort are shown in Table 1.

**Table 1:** Patients’ Demographic Characteristics

Characteristic	No. (%)
Sample size	145
Sex	
Male	83 (57.24%)
Female	62 (42.76%)
Age	
Median (interquartile range)	56 (46‐67)
Range	7‐88
Diagnosis	
Lung cancer	25 (17.24%)
Interstitial lung disease	56 (38.62%)
Other lung disease	35 (24.14%)
Control group	29 (20.00%)

Note

Since the patients' age distribution is non‐normal, expressed in interquartile range.

### 2.2 Patient enrollment criteria

All patients with ILD fulfilled the 2013 American Thoracic Society/European Respiratory Society (ATS/ERS) classification criteria for ILD, excluding malignant tumors, infections, and other lung diseases. The inclusion criteria for patients with lung cancer were surgical or pathological biopsy with no ILD. Among the enrolled patients with other respiratory diseases, those with chronic obstructive pulmonary disease were enrolled according to the diagnostic criteria for the 2017 Guidelines for the Diagnosis and Treatment of Chronic Obstructive Pulmonary Disease, without mental illness, severe heart and liver and kidney disease, active tuberculosis, and respiratory failure. The criteria for bronchodilation were confirmed by HRCT and absence of cystic fibrosis, active tuberculosis, severe pneumonia, and severe heart disease.

### 2.3 Measurement of KL‐6 level

Blood collection was performed following a standard protocol. Peripheral blood samples were collected from each patient using a vacuum blood vessel containing separating gel. After centrifuging for 10 minutes at 1000 × *g*, the upper layer was collected for testing. Prior to testing, the serum was kept at room temperature for 30 minutes and was agitated in a vortex mixer. The serum fractions were aliquoted in 1.5 mL Eppendorf tubes and stored at 4°C until analysis. Repeated freeze‐thaw cycles were avoided.

We evaluated the basic performance of KL‐6 assays using SYSTEM A, a fully automated immunochemistry analyzer that employs a CLEIA methodology with a two‐step sandwich immunoassay. The primary antibody was biotin‐binding anti‐KL‐6 mouse monoclonal antibody, and the secondary antibody was alkaline phosphatase (ALP)‐labeled anti‐KL‐6 mouse monoclonal antibody. As a control method, CLEIA on SYSTEM B was performed according to the manufacturer's instructions. SYSTEM B is a fully automated CLEIA. All assays relay on two m‐Ab, one labeled with ALP and the other one coated on iron beads. Chemiluminescence is produced after 3‐(2′‐spiroadamantane)‐4‐methoxy‐4‐(3″‐phosphoryloxy)‐phenyl‐1,2‐dioxetane (AMPPD) hydrolysis by ALP into an unstable product that stabilizes by emitting light, measured at 477 nm.^14^ The analytical measurement ranges of SYSTEM A and SYSTEM B were 10‐6000 U/mL and 50‐10 000 U/mL, respectively. The cutoff value for KL‐6 was 500 U/mL in all two systems.

### 2.4 Ethical approval

This study and the use of the human serum samples were approved by the Ethics Committee of The First Affiliated Hospital of Guangzhou Medical University (Ethics—[2017]—Reagents—35‐02).

### 2.5 Data analysis

Statistical analyses were conducted using Excel 2016 (Microsoft Excel^®^ 2016) and SPSS 22.0 (IBM Corp.). Parametric quantitative data were presented as mean ± standard deviation. Nonparametric quantitative data were presented as median (interquartile range). Consistency between the two systems was evaluated using sensitivity, specificity, positive predictive value, negative predictive value, and kappa value. Disease diagnosis was used as the gold standard to establish the receiver operating characteristic (ROC) curve, and scatter plots and Bland‐Altman plot were used to demonstrate the concentration distribution in the two methods. Correlation analyses for nonparametric data were performed using the Spearman tests, with the correlation coefficients presented as “*r*_S_,” and the closer the *r*_S_ value is to −1 or +1, the stronger the correlation. And intraclass correlation coefficient (ICC) was used to evaluate the repeatability or consistency of the two systems. Mann‐Whitney *U* test and Kruskal‐Wallis *H* test were used to determine the difference in KL‐6 level among two or multiple groups, respectively. Moreover, Bonferroni correction was used to adjust the level of significance after the two‐by‐two comparison. A *P*‐value < .05 was considered statistically significant.

## 3 RESULTS

### 3.1 Consistency and correlation between the two systems

**Figure 1:**
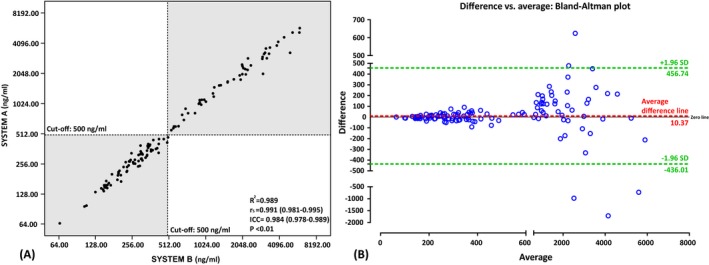
A, A scatter plot and (B) Bland‐Altman plot based on the KL‐6 titers detected by two systems. SYSTEM A, LUMIPULSE G1200 analyzer; SYSTEM B, HISCL‐5000 analyzer. “*R* ^2^” represents the linear coefficient of the fitting curve of the two indexes, and “*r*_S_” represents the correlation coefficient of the Spearman correlation analysis. The lines in the scatter plot are the cutoff values of the two systems. In the (B) figure, the black line represents the zero line, red dashed line represents the average difference value, and the upper and lower two green dashed lines represent 95% limits of agreement

The two systems simultaneously detected serum KL‐6 index in the above‐mentioned patients and performed consistency analysis. With a cutoff value of 500 U/mL as the diagnostic threshold, the qualitative agreement rate of the two systems is 99.20%, and the sensitivity, specificity, and kappa value were 100%, 98.63%, and 0.984 (95% CI, 0.952‐1.000), respectively (Table 2). By drawing the scatter diagram, we can directly show the distribution of the KL‐6 level detected by the two systems. From the results of Figure 1, we can observe that there is a very good linear correlation between the two systems (*R* ^2^ = 0.989), and the results of the Spearman correlation analysis also show that there is a strong correlation between serum KL‐6 levels measured by the two systems (*r*_s_ = .991 [95% CI, 0.981‐0.995], *P* < .001). Based on the estimates of single measures, we determined the intraclass correlation coefficient of the diagnostic test repeatability evaluation was 0.984 (95% CI 0.978‐0.989; *P* < .001). As can be seen from the Bland‐Altman plot, 4.00% (5/125) of the points were outside 95% limits of agreement (LoA; −436.01 to 456.74). Within the consistency limit, the maximum absolute value of the difference between the KL‐6 value measured by SYSTEM A and SYSTEM B was 1715 mg/mL, and the average value of the difference was 10.37 mg/mL.

**Table 2:** Evaluation of the consistency of KL‐6 detected by LUMIPULSE G1200 analyzer and HISCL‐5000 analyzer

		SYSTEM A (ng/mL)		CO	SE	SP	PPV	NPV	Kappa (95% CI)	*r*_S_	ICC
		≤500	>500								
SYSTEM B (ng/mL)	≤500	72	0	99.20%	100.00%	98.63%	98.11%	100.00%	0.984 (0.952‐1.000)	.991**	0.984**
	>500	1	52								

Note

The consistency of the SYSTEM B was evaluated using the SYSTEM A as a reference method. And 500 U/mL is used as the cutoff value of the KL‐6.

Abbreviations: CO, consistency; ICC, intraclass correlation coefficient; NPV, negative predictive value; PPV, positive predictive value; rS, Spearman's rho; SE, sensitivity; SP, specificity; SYSTEM A, LUMIPULSE G1200 analyzer; SYSTEM B, HISCL‐5000 analyzer.

** Correlation is significant at the 0.01 level (2‐tailed).

**Figure 2:**
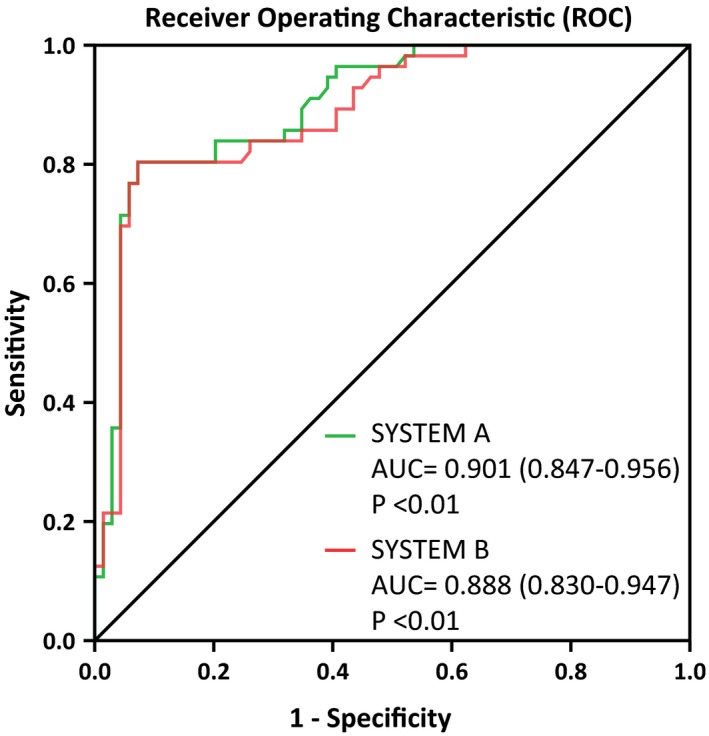
Relative operating characteristic curve. SYSTEM A, LUMIPULSE G1200 analyzer; SYSTEM B, HISCL‐5000 analyzer; AUC, area under the curve

Additionally, the results of the disease diagnosis were used as reference standard to evaluate the clinical diagnostic efficacy of the two systems. The consistency of SYSTEM B was 85.60%, while that of SYSTEM A was 86.21%. Furthermore, the consistency evaluation indexes of the two systems are >80%, indicating that the clinical diagnostic effectiveness of the two systems is highly consistent. The kappa values were 0.707 (95% CI, 0.582‐0.832) and 0.707 (95% CI, 0.588‐0.826) in SYSTEM B and SYSTEM A, respectively. As shown in Table 3, the ROC curve was used to compare the differences in diagnostic performance between the two systems. Among these, 56 patients were diagnosed by disease in the ILD group and 89 patients in non‐ILD group. The areas under the ROC curve were 0.901 (95% CI, 0.847‐0.956) and 0.888 (95% CI, 0.830‐0.947) in SYSTEM A and SYSTEM B, respectively (Figure 2). The difference in areas under the ROC curve between the two systems was 0.013, and the z‐statistic was 1.772. The difference in diagnostic value between the two systems was not statistically significant (*P* = .0763).

**Table 3:** The clinical diagnostic performance of KL‐6 detected by LUMIPULSE G1200 analyzer and HISCL‐5000 analyzer

		ILD		CO	SE	SP	PPV	NPV	Kappa (95% CI)	AUC (95% CI)
		Positive	Negative							
SYSTEM A (ng/ml)	≤500	62	11	85.60%	80.36%	89.86%	86.54%	84.93%	0.707 (0.582‐0.832)	0.901 (0.835‐0.947)
	>500	7	45							
SYSTEM B (ng/ml)	≤500	80	11	86.21%	80.36%	89.89%	83.33%	87.91%	0.707 (0.588‐0.826)	0.888 (0.820‐0.938)
	>500	9	45							

Note

Analyze the clinical diagnostic performance of the two analyzers with the exact diagnosis of the disease as a gold standard and compare the differences between the two analyzers.

Abbreviations: AUC, area under the curve; CO, consistency; ILD, interstitial lung disease; NPV, negative predictive value; PPV, positive predictive value; SE, sensitivity; SP, specificity; SYSTEM A, LUMIPULSE G1200 analyzer; SYSTEM B, HISCL‐5000 analyzer.

### 3.2 Diagnostic performance of Kl‐6 detected by SYSTEM A in ILD

**Figure 3:**
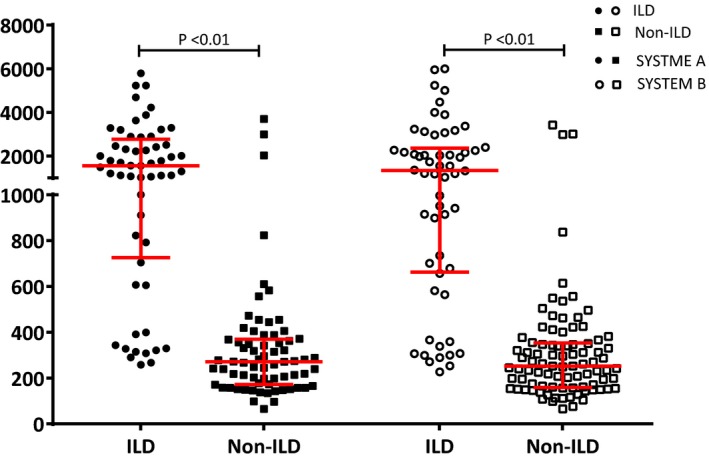
Serum KL‐6 level in the ILD and non‐ILD groups. ILD, interstitial lung disease; SYSTEM A, LUMIPULSE G1200 analyzer; SYSTEM B, HISCL‐5000 analyzer. Nonparametric quantitative data were presented as median (interquartile range). *P*‐values were calculated using Mann‐Whitney *U* test between two groups

Figure 3 shows the distribution of serum KL‐6 level in SYSTEM A and SYSTEM B among the various subject groups, ILD and non‐ILD groups (including patients with lung cancer and other respiratory diseases and healthy controls). In SYSTEM A, the KL‐6 level in the ILD group (1339.5, 662.5‐2363) was significantly higher than that in the non‐ILD group (252, 158.5‐353; Mann‐Whitney *U* = 381.5, *P* < .01). Moreover, the same result is true in SYSTEM B. The KL‐6 level (1558, 726‐2772.5) was significantly higher in the ILD group than that in the non‐ILD group (271, 172‐369.5; Mann‐Whitney *U* = 492.0, *P* < .01).

**Figure 4:**
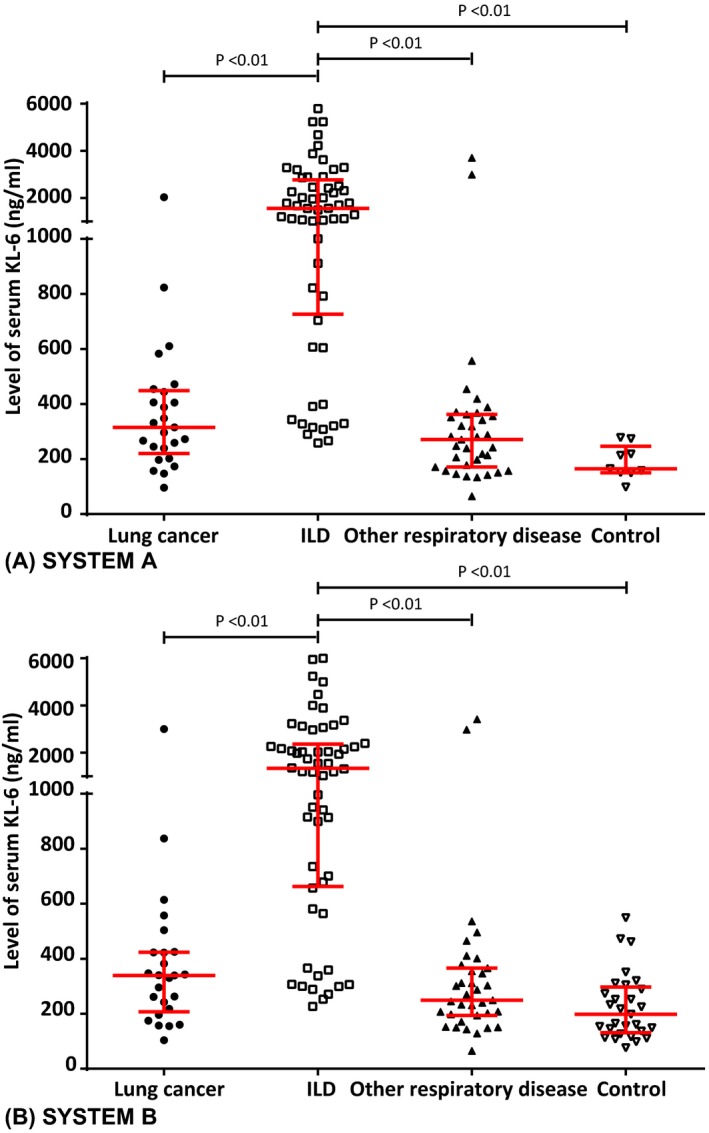
Serum KL‐6 levels in the lung cancer, ILD, other respiratory disease, and healthy control groups. ILD, interstitial lung disease; A, SYSTEM A, LUMIPULSE G1200 analyzer; B, SYSTEM B, HISCL‐5000 analyzer. Nonparametric quantitative data were presented as median (interquartile range). *P*‐values were calculated using Kruskal‐Wallis *H* test among the four groups

Kruskal‐Wallis *H* test was used to determine differences in KL‐6 levels in the ILD, lung cancer, other lung disease, and control groups. Figure 4A showed that the KL‐6 level (1558, 726‐2772.5) in the ILD group detected by SYSTEM A was significantly higher than that in the lung cancer group (339, 207‐424), other respiratory disease group (249, 194‐366), and control group (198, 131.5‐297; Kruskal‐Wallis *H* = 63.19, *P* < .01), but there was no statistically significant difference between the lung cancer, other respiratory disease, and control groups. In SYSTEM B, the KL‐6 level in the ILD group (1558, 726‐2772.5) was also significantly higher than those in the lung cancer group (315, 220.5‐449), other respiratory disease group (271, 171‐362), and control group (165, 150‐246; Kruskal‐Wallis *H* = 71.64, *P* < .01), but there was no statistically significant difference between the three groups (Figure 4B).

## 4 DISCUSSION

In our study, we compared the KL‐6 levels in 145 serum samples between SYSTEM A and SYSTEM B. The results showed that serum KL‐6 in SYSTEM A had acceptable sensitivity and specificity and was comparable to that in SYSTEM B. Overall, we found a high degree of agreement among the two systems (agreement, 99.20%). Compared with SYSTEM B, SYSTEM A has a lower minimum detection limit (10 U/mL) and wider range of low detection values.

In this study, 56 patients who were diagnosed with ILD were selected. Other patients with lung cancer and other respiratory diseases, and healthy individuals were included for comparison. With a cutoff level of 500 U/mL, the results showed that serum KL‐6 levels in patients with ILD were significantly higher than those in other groups. Therefore, high serum KL‐6 levels were useful in the adjunctive diagnosis of ILD.^15^, ^16^

In our study, the diseases in the ILD group included connective tissue–associated interstitial pneumonia, autoimmune characteristics of interstitial pneumonia, idiopathic pulmonary fibrosis, vasculitis‐related interstitial pneumonia, smoking‐related interstitial pneumonia, and allergic reaction alveolitis. Using the recommended cutoff level (500 U/mL) in the monitoring system as reference, the patient's serum KL‐6 test has a good positive rate. Due to the small sample size in some ILD subcategories, there is no difference in the KL‐6 level in each of the subcategories (the result was not shown), and more cases need to be accumulated for further study. Although the KL‐6 level does not distinguish the subtypes of ILD, KL‐6 showed a high level of diagnosis for ILD subtypes on the premise of other indicators. Since this is a noninvasive test, KL‐6 test has high clinical application value.

Hu et al^17^ reported that, in China, when the cutoff value was set to 500 U/mL, the sensitivity and specificity of KL‐6 in ILD diagnosis were 77.75% and 94.51%, respectively. However, recently, literature reports have shown that the cutoff value of serum KL‐6 level varies among different races.^18^, ^19^ This study uses the recommended detection cutoff value (500 U/mL) of the KL‐6 kit produced in Japan as a reference, which may have an impact on the diagnosis of ILD. Therefore, it should establish its own reference interval in subsequent experiments.

It has been reported that KL‐6 has increased expression in various malignant tumors, can be used as a potential biomarker for tumors, and is of great value in the diagnosis, treatment, and monitoring of tumors.^20^ However, in this study, the serum KL‐6 level in patients with lung cancer was not significantly increased compared with that in healthy individuals, probably because KL‐6 was not adequately specific in lung cancer, and in clinical practice, the sensitivity and specificity should be improved by combining it with other tumor markers.

There are several limitations in our study. Although the serum KL‐6 levels in 56 patients with ILD were compared, the sample size was relatively small. If the sample size is increased, the results will be more representative. There is an age difference between the experimental group and healthy individuals due to the prevalent elderly population in the ILD group. If age‐appropriate subjects are included as much as possible, the diagnostic value of the test results will be more credible. This study did not investigate the correlation between KL‐6 and pulmonary function, HRCT, and drug administration in patients. No artificial intervention was conducted on the treatment of patients to reduce the influence of other confounding factors. Because the cutoff value of the KL‐6 level in patients with ILD was not determined, the correlation between KL‐6 level and clinical activity of ILD was not obtained. In the next step, a multicentre large‐scale study can be conducted to refine the classification of patients with ILD, explore changes in KL‐6 levels among subclass diseases, and design more rigorous experiments for further clarification.

## 5 CONCLUSION

The HISCL‐5000 CLEIA system has a high diagnostic efficiency. The method can be applied to the quantitative detection of serum KL‐6 in patients with respiratory diseases. Compared with those in other respiratory diseases, the serum KL‐6 level in patients with ILD is significantly increased, suggesting that clinicians can use KL‐6 in the auxiliary diagnosis of ILD.
